# Bioactive Glass: Methods for Assessing Angiogenesis and Osteogenesis

**DOI:** 10.3389/fcell.2021.643781

**Published:** 2021-06-14

**Authors:** Jos Crush, Ali Hussain, K. T. M. Seah, Wasim S. Khan

**Affiliations:** Division of Trauma and Orthopaedic Surgery, University of Cambridge, Cambridge, United Kingdom

**Keywords:** bioglass, angiogenesis, osteogenesis, bioactive glass, regenerative

## Abstract

Biomaterials are playing an increased role in the regeneration of damaged or absent bone tissue in the context of trauma, non-union, infection or congenital abnormality. Restoration of not only the physical scaffold that bone provides, but also of its homeostatic functions as a calcium store and hematopoietic organ are the gold standards of any regenerative procedure. Bioactive glasses are of interest as they can bond with the host bone and induce further both bone and blood vessel growth. The composition of the bioactive glasses can be manipulated to maximize both osteogenesis and angiogenesis, producing a 3D scaffolds that induce bone growth whilst also providing a structure that resists physiological stresses. As the primary endpoints of studies looking at bioactive glasses are very often the ability to form substantial and healthy tissues, this review will focus on the methods used to study and quantify osteogenesis and angiogenesis in bioactive glass experiments. These methods are manifold, and their accuracy is of great importance in identifying plausible future bioactive glasses for clinical use.

## Introduction

The regeneration of damaged or absent bone tissue in the context of trauma, non-union, infection or congenital abnormality remains a challenging task for medical teams around the world. Restoration of not only the physical scaffold that bone provides, but also of its homeostatic functions as a calcium store and hematopoietic organ are the gold standards of any regenerative procedure.

The incidence of bone defects is increasing due in part to an aging population (Amini et al., 2012), and the need for bone regeneration in these situations has led to the development of multiple techniques that aim to restore function and structure, such as, autologous bone grafting, allografting and prostheses. These techniques are limited by procedural issues, shortage of suitable tissue grafts, and the inability to fully replace the function of the tissue (Dimitriou et al., 2011). For example, with an aging patient demographic of patients who may suffer increasingly from osteoporosis, donor sites may not provide suitable bone graft material.

More recently, focus has changed to the development of biomaterials. These are engineered materials designed to induce bone regrowth and regeneration. There is now a dedicated field to developing biomaterials for bony defects using various materials, production methods and scaffold designs with the option to embed biological materials such as cells or growth factors within them.

**FIGURE 1:**
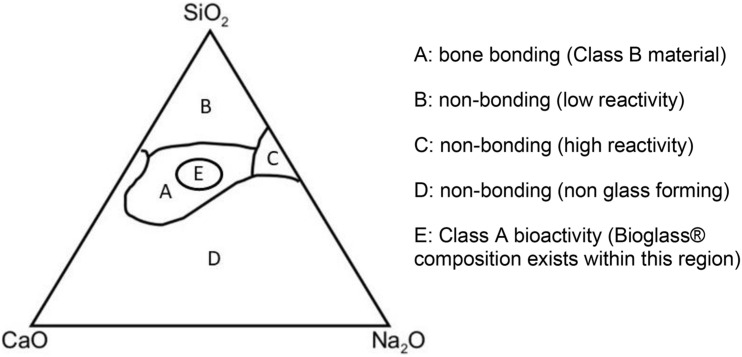
Compositional diagram for bone bonding. Class A biomaterials (Region E) are able to recruit cells involved in bone formation while Class B biomaterials only demonstrate bone growth at the implant-bone interface (Region A). SiO_2_, silicon dioxide; CaO, calcium oxide; Na_2_O, sodium oxide.

One such group of materials is the now well-established bioactive glasses. These glasses are bioactive as they bond with the host bone and induce further bone and blood vessel growth. The original product is the now trademarked 45S5 “bioglass^®^” designed in the late 1960s; a silicon-based glass network with Na_2_O, CaO, and P_2_O_5_ network modifiers (due to trademark, bioglass^®^ refers to the original 45S product only, all other materials are hence known as bioactive glasses) (Hench et al., 1971). Since then, there have been other bioactive glass compositions (Figure 1) each with various strengths and weaknesses (Hench, 1991; Brauer, 2015). Despite encouraging data, only a few products have been licensed for clinical use including middle ear implants, moldable granules, and toothpastes (Jones, 2013).

As well as modifications to the composition of the bioactive glasses in an effort to maximize bone and blood vessel growth, there is also vast interest in different manufacturing techniques that allow production of other desirable characteristics, such as porosity, pore interconnectivity and overall strength (Jones et al., 2006). This has led to the idea of building 3D scaffolds that induce bone growth whilst also providing structures that resist physiological stresses (O’Brien, 2011; Fiume et al., 2018).

Throughout the literature studying bioactive glasses *in vivo* and *in vitro*, the primary endpoint is very often the ability to form substantial and healthy new bone tissue (Ranmuthu et al., 2020). This necessarily requires formation of both new bone and new blood vessels (osteogenesis and angiogenesis, respectively). This review will focus on the methods used to study and quantify osteogenesis and angiogenesis in bioactive glass experiments. These methods are manifold, with little experimental consistency across the literature. Their accuracy is therefore of great importance in identifying plausible future bioactive glasses for clinical use.

## A Brief Background and Current State of Play

Prior to the production of the first bioactive glass (45S5 Bioglass^®^) in 1969, biomaterials were designed to be as inert as possible so that they replaced the tissue, rather than regenerated it (Hench, 2006). This meant the implant was designed to be structurally very rigid, chemically inert and compressed into the defect, fitting by interference. Subsequently, a lack of chemical bonding led to scar tissue formation around the implant and the accumulation of load and microforces on the scar tissue-implant interface resulted in implant failure. These are known as inert, or first-generation biomaterials.

Bioglass^®^ was designed to be both biologically inert and able to chemically bond with bone. This lead to the now well-known result that the glass could not be removed from the bone without breaking the bone itself (Hench et al., 1971). This material provided not only the first example of a second-generation, but also of a third-generation biomaterial, in that it not only chemically bonds to bone, but also induces bone growth (Hench, 2013).

The mechanism of bone bonding is discussed later in this article, but relies on the surface of the glass forming an HCA network after dissolution of various glass ions, which also stimulate cells to form collagen networks. Surface area is therefore a key characteristic of the product as it affects dissolution.

Since then, the original Bioglass^®^ composition has found some commercial success, particularly in dentistry. Varying compositions of network modifiers in silicone glasses have been trialed but bar one, none are as bio reactive as the original (Jones, 2013). There has also been interest in non-silicate glass forms such as borate or phosphate based glasses, and generally bioactive glasses are classified by their network forming compound. These glasses can then be further characterized by the network modifiers and by the manufacturing process and final physical form they take.

The products produced for clinical use have been developed from monoliths to granulates with sizes as small as 18micrometres and putty forming substances that allow surgeons to inject the biomaterial into the defect and fill it completely (Rust et al., 1996).

Despite the bioactivity of 45S5 bioglass, its inherent chemical composition leads to limitations in the structures can be manufactured from it. In particular during the traditional manufacturing method of melt quenching, the glass tends to crystallize during the sintering phase, producing regions of crystal-amorphous transition which are prone to fracture. It is therefore not possible to build modeled scaffolds from this particular bioactive glass (though this has been achieved with newer bioactive compositions). Formation of scaffolds is desirable for both their structural integrity and vastly increased surface area, and is the current focus of much of the field of biomaterials (Jones et al., 2006). The advent of new manufacturing techniques such as sol-gel processing has provided a solution; glasses have been produced with increased porosity and therefore surface area for bone formation (Hench, 1997). This technique can produce nanoporous powders or monoliths (Lin et al., 2009). As this technique does not require heating to the same degree as traditional quenching techniques, the composition of the glasses tends to be simpler.

More recently, certain quench derived bioactive glasses have been produced which combined with novel manufacturing ideas such as composites and nanoparticle or nanofiber formation aim to produce an ideal mix of structural integrity and bone-induction in a bioactive glass scaffold (Wu and Chang, 2012; Jones, 2013; Ranmuthu et al., 2020). The porosity of the scaffold plays a crucial role in inducing tissue regeneration and varies between *in vitro* and *in vivo* models, with improved osteogenesis seen with higher porosity and pore size *in vivo* (Karageorgiou and Kaplan, 2005). Combining bioactive glass with other biomaterials in different ratios allows their properties to be tailored to fit their need. These composites can be 3D-printed to generate specific architecture and in combination with other techniques, create optimized porosity for facilitating angiogenesis and osteogenesis (Gomez-Cerezo et al., 2021). These scaffolds also allow concepts such as drug or growth factor delivery to be realized. Altering the bioactive glass composition in composites has been shown to modulate the local release of drugs such as vancomycin (Kim et al., 2005), which has promising implications for the treatment of bone infection. The structure and manufacturing processes of these various glasses is reviewed in detail elsewhere (Jones, 2013).

In addition, bioactive glass also has the potential to be used more widely in tissue repair due to its ability to induce angiogenesis. Bioactive glass improved the mechanical properties and scaffold bioactivity when added to poly(glycerol sebacate), an elastomeric polymer studied for cartilage repair (Souza et al., 2017). The regeneration of multiple tissue types may also be facilitated by the use of multi-layered, functionally graded scaffolds (Baino et al., 2018).

This summary outlines the current state of play for bioactive glass design. This field has multiple avenues of research and understanding the techniques that allow us to assess osteogenesis and angiogenesis is paramount.

## An Overview of Bone and Blood Vessel Growth in Bioactive Glass

**FIGURE 2:**
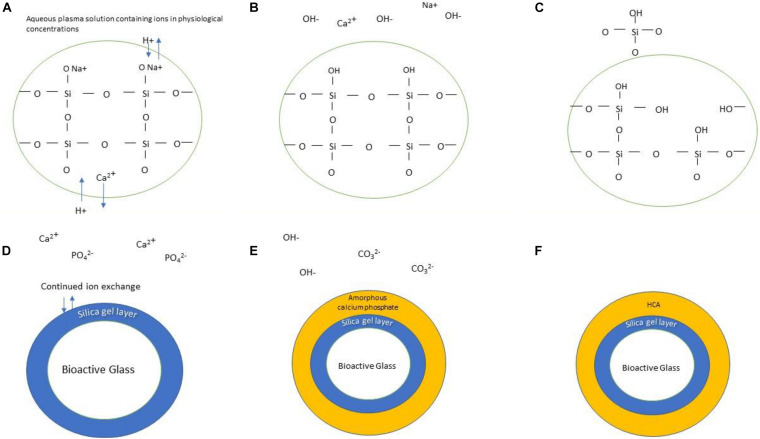
Schematic illustration of the formation of HCA layer on the surface of bioactive glass. The stages are as follows: **(A)** Bioactive glass is submerged in physiological solution; Ionic exchange occurs between the doped ions and protons in the solution, forming hydroxyl groups **(B)**. Continuation of this process releases silicon in the form of silanol **(C)**. A silica gel layer is formed **(D)** which allows continued ion exchange of calcium and phosphate groups, forming an amorphous calcium phosphate layer atop the silica gel layer **(E)**. Continued incorporation of hydroxyl and carbonate groups results in the formation of an HCA layer **(F)**.

A key part of the design criteria of bioactive glasses is the ability to bond with bone. As well as this, they can ideally induce its growth not only at the bone-material interface, but also away from the implant site. These are features shown by the original Bioglass^®^ and represent the gold standard. The glass bonds with bone in two basic steps, first, formation of a hydroxyl carbonated apatite (HCA) layer on the glass surface and second, the subsequent cellular responses to this both in the local area and more distally (Figure 2). The mechanisms responsible for the latter are less well-understood and are an active area of research.

Following the generation of the HCA layer, proteins and subsequently osteoprogenitor cells are attracted to the bone surface and begin forming extracellular matrix (ECM) (Hench, 2013). Ionic dissolution products from the bioactive glass regulate the proliferation and differentiation of the osteoprogenitors. The specific concentration of Si and Ca ions from the bioglass is important in maximizing ECM production (Hench, 2009; Hoppe et al., 2011). Seemingly the ionic dissolution products lead to upregulation in several gene families involved in bone production *in vitro* (Xynos et al., 2001).

The mechanism of angiogenesis in these constructs has also been studied, but not as extensively. This small section will focus on the perceived mechanism of growth in glasses not containing additional cellular components. As with bone growth, ionic dissolution products are likely responsible for the angiogenic potential, and there is much interest in the relative concentrations and dissolution rates of each of these.

The proposed mechanisms are complex and not well understood. It is postulated that silicon ions mimic hypoxic conditions, acting both directly on fibroblast and or osteoblasts to increase vascular endothelial growth factor (VEGF) release (Xynos et al., 2000), which in term activates the downstream angiogenesis pathways (Dashnyam et al., 2017). This may well be via interaction with the hypoxia inducible factor (HIF) pathway, which is the mechanism by which hypoxia drives vascular growth in tumors, and also plays an important role in physiological bone growth (Kong et al., 2014). It must also be remembered that the same factors that drive angiogenesis will affect osteogenesis both directly and indirectly by the provision of elements for new bone growth, and by acting directly on the osteoprogenitors. It is a physiological necessity that these are paired processes, as diffusion alone limits delivery of provisions for cellular metabolism to within 2–300 μm of a blood vessel (Jain et al., 2005). The interaction between the two processes in bioactive glass constructs is complex and worthy of its own library of research (Grosso et al., 2017). It should be noted that with some constructs there is the option of implanting cell lines or growth factors, which may change the mechanisms of growth.

The techniques used fall broadly into the following categories: histological staining and immunohistochemistry, imaging, gene expression and growth factor assays, enzymatic activity assays and spectroscopy techniques. This article will focus on the commonly used techniques.

In the formation of any new tissue, angiogenesis must occur simultaneously to overcome the limitations of diffusion alone. There is evidence to suggest bioactive glasses can induce angiogenesis particularly well when compared to other bioactive materials, these properties are obviously important for both bone and soft tissue repair and have been reviewed extensively (Gorustovich et al., 2010).

## Light Microscopy: Histology and Immunohistochemistry (IHC)

The method of analysis favored by early groups is the direct examination of bone structure or blood vessel number and diameter by histological examination. The tissue of interest is fixed and subsequently decalcified to allow sections to be cut from the specimen. A stain or antibody is applied to the section which is examined with light microscopy. Although mentioned here, non-IHC staining is seldom used in current studies and IHC tends to be favored.

Histological techniques have the benefit of being able to directly examine the volume and quantity of vessel or bone growth without being hindered by image resolution or contrast perfusion as in radiological techniques. They also permit a direct qualitive analysis of the vascular and bone structures, with analysis of their makeup possible through the various histological features present. This means that tissue morphology can be compared to “normal” sections prepared in the same way.

Plain light microscopy using traditional staining will allow for a structural and numerical comparison of the new bone and vessels across various specimens. To some degree, cell types are able to be recognized, but there are no markers of biological activity in this type of staining.

Valuable information can still be obtained from these techniques when assessing samples for osteogenesis. Comparing the histological appearance of several test groups can provide information on the amount of bone formation, the presence of immature or lamellar bone and amount of bioactive glass degradation. This can be particularly useful when assessing whether changes to a bioactive glass scaffold affects the induction of mature bone at defect sites. The yield of these techniques can be improved by fluorescence labeling. Use of fluorescent dyes such as alizarin red S, calcein and tetracycline localize to areas of high calcium and can also aid in differentiating newly formed bone from existing bone. Table 1 shows some of the commonly used stains which are relevant to osteogenesis and angiogenesis.

**TABLE 1:** Common stains used in assessing osteogenesis and angiogenesis.

Stain	Target	Description
Haematoxylin	Stains nuclei blue	Stains the chromatin in cell nuclei dark blue. Also stains rough endoplasmic reticulum, ribosomes, collagen, myelin, elastic fibers, and mucins.
Eosin	Stains cytoplasm pink	Often used as a counterstain with haematoxylin, together known as H&E. Stains cytoplasm pink.
Masson’s trichrome	Stains collagen blue/green	Variable three color staining depending on the specific application of the stain. Usually produces: blue/green collagen; red keratin and muscle fibers; pink cytoplasm and black nuclei.
Toluidine blue	Stains proteoglycans and glycosaminoglycans purple	Stain color is produced by metachromasia. High affinity for DNA and RNA which are stained blue. Stains proteoglycans and glycosaminoglycans purple.
Van Gieson	Stains collagen red	Combination of piric acid and acid fuchsin. Differentiates collagen from other connective tissue. Known as HvG when combined with haematoxylin and collagen will appear pink.
Alizarin Red S	Stains calcium orange/red	Used to locate tissues with high calcium content such as bone
Calcein	Binds to calcium ions	Fluoresces green with excitation/emission wavelengths 488 nm/520 nm, respectively.
Tetracycline	Binds to calcium	Fluoresces yellow with excitation/emission wavelengths 450–490 nm/529 nm. Localizes to sites of active mineralization.

IHC retains all of the beneficial features of traditional histological stains, but with the added ability to assess biological activity in the cells, and better recognize complex cell lines. This type of analysis is used commonly in assessing angiogenesis in bioactive glass experiments as it overcomes issues that cross-sectional imaging has with imaging vasculature. It is less commonly used to assess osteogenesis where CT imaging prevails. Table 2 shows some of the common IHC targets used to assess angiogenesis and osteogenesis.

**TABLE 2:** Common IHC targets used to quantify angiogenesis and osteogenesis.

IHC target	Marker of	Description
CD31/PECAM-1	Angiogenesis	Platelet endothelial cell adhesion molecule (PECAM-1), or CD31, is expressed by endothelial cells, platelets and all leucocytes. It is used as a biomarker for the presence of epithelial cells and for angiogenesis.
CD34	Angiogenesis	Expressed in haematopoietic stem cells. Used as a biomarker for vascular endothelial cells to assess angiogenesis (Nielsen and McNagny, 2008).
α-SMA	Angiogenesis	Alpha-Smooth Muscle actin is highly expressed in vascular smooth muscle cells which facilitates its use as a biomarker of angiogenesis.
VEGF	Angiogenesis	Vascular endothelial growth factor (VEGF) is a potent simulant of angiogenesis. It would be expected to be upregulated in regions undergoing active angiogenesis. It also has effects on bone remodeling with pro-migratory and pro-proliferative effects on osteoblasts and stimulates osteoclasts via the RANK pathway (Yang et al., 2012)
Endomucin	Angiogenesis	A marker of endothelial and haematopoietic stem cells.
ALP	Osteogenesis	ALP is expressed early in bone development and has is involved with the early stages of calcification and mineralization.
Osteopontin	Osteogenesis	Osteopontin (OPN) is a non-collagenous protein component of extracellular bone and is expressed early in osteogenesis.
Type I collagen	Osteogenesis	Type I collagen is a mid-to-late target in investigating osteogenesis. It is the predominant protein in osteoid.
Osteocalcin	Osteogenesis	Produced exclusively by osteoblasts and a major non-collagenous component of bone. Its expression peaks late in osteogenesis.

Several IHC protein targets are used to evaluate bioactive glass angiogenic and osteogenic activity. For example, having targets that peak in expression during different stages of osteogenesis provides additional information on the scaffold’s suitability for defect repair. An ideal bioglass scaffold will result in adequate stimulation of osteogenesis and will promote progression to mature bone. An appreciation for the temporal difference in expression is needed when timing collection of samples and selection of IHC targets.

Early targets include alkaline phosphatase (ALP) and osteopontin (OPN). ALP is expressed early in bone development and has functions in initiating calcification and mineralization while OPN is an extracellular structural component of bone. It has a multifaceted role during early osteogenesis including playing a key role in influencing MSCs toward an osteogenic lineage (Chen et al., 2013). In addition, its expression is upregulated during the pro-osteogenic differentiation of these cells. Its role and high expression in early osteogenesis makes it a useful target in IHC techniques when assessing the effectiveness of a bioglass scaffold. Type I collagen (COL1) is a mid-to-late target in osteogenesis. During osteogenesis, osteoblasts secrete osteoid which is then mineralized to become mature bone. The predominant component of osteoid is type I collagen. Osteocalcin (OCN) is produced by osteoblasts and, like OPN, is another non-collagenous protein component of bone matrix; however, its expression peaks late in osteogenic differentiation.

However, these methods have several disadvantages. Histological analysis means that longitudinal studies are seldom possible on the same subject, which introduces more variation and necessitates more animal subjects. The nature of histological sections means they are a 2D representation of a 3D structure, and only a “snapshot” of the overall structure. This can be overcome in part using serial sections and analyzing them as a cohort, however, as mentioned latterly, truly quantitative analysis of bone or blood vessel growth is not possible.

## Imaging

Imaging in this field is dominated by microCT due to its high contrast and spatial resolution. In particular, the technique is useful in assessing bone structure, with more recent advances made in its ability to image vascular structures by using contrast media (e.g., most commonly iodine-based agents such as Omnipaque and Isovue). Other radiological techniques such as nuclear imaging (i.e., single photon emission computed tomography and positron emission tomography) have also been used either alone or in combination with CT imaging (Fragogeorgi et al., 2019).

### MicroCT

MicroCT finds its origins in the early 1980s as a research tool which afforded higher quality imaging than conventional CT, but at the cost of greater radiation doses. It was not until 1994 that this became a viable option with commercially available scanners, allowing microCT to become the mainstay of bone imaging in animal models. Future developments in this imaging modality are discussed at the end of this section.

In principle a microCT scanner is no different to a conventional CT scanner, using the attenuation of X-ray radiation in multiple 2D sections to produce a 3D image of an object through back projection. It is able to produce voxels of <1 μm, where trabeculae in mice models are around 30 μm at their smallest (Martín-Badosa et al., 2003).

Live specimens are imaged using a scanner that rotates around the animal, and prepared bone specimens are themselves rotated. Live specimens must be anaesthetized to minimize movement artifact and to position them optimally and reliably with respect to the radiation source. This is particularly important when considering image registration which is discussed later (Campbell and Sophocleous, 2014). Ex-vivo specimens are imaged in a medium of PBS, ethanol or formalin which reduces the contrast between the medium and the bone. It is vital that the positioning of the specimen and the medium used are constant across the study.

#### Osteogenesis

This modality produces 3D images of the bone microstructure as well as quantified measures of bone morphometry and tissue density. The output morphometric data and their descriptions are illustrated in Table 3, and the most commonly used are bone volume, BMD and bone volume fraction.

**TABLE 3:** Definitions and descriptions of bone morphometric data.

Abbreviation	Variable	Description	Standard unit
TV	Total volume	Volume of the entire region of interest	mm^3^
BV	Bone volume	Volume of the region segmented as bone	mm^3^
BS	Bone surface area	Surface of the region segmented as bone	mm^2^
**BV/TV**	**Bone volume fraction**	Ratio of the segmented bone volume to the total volume of the region of interest	%
BS/TV	Bone surface density	Ratio of the segmented bone surface to the total volume of the region of interest	mm^2^/mm^3^
BS/BV	Specific bone surface	Ratio of the segmented bone surface to the segmented bone volume	mm^2^/mm^3^
Conn.D	Connectivity density	A measure of the degree of connectivity of trabeculae normalized by TV	1/mm^3^
SMI	Structure model index	An indicator of the structure of trabeculae; SMI will be 0 for parallel plates and 3 for cylindrical rods	-
**Tb.N**	**Trabecular number**	Measure of the average number of trabeculae per unit length	1/mm
**Tb.Th**	**Trabecular thickness**	Mean thickness of trabeculae, assessed using direct 3D methods	mm
**Tb.Sp**	**Trabecular separation**	Mean distance between trabeculae, assessed using direct 3D methods	mm
Tb.Th.SD	Standard deviation of trabecular thickness	Measure of the homogeneity of trabecular thickness, assessed using direct 3D methods	mm
Tb.Sp.SD	Standard deviation of trabecular separation	Measure of the homogeneity of trabecular separation, assessed using direct 3D methods	mm
DA	Degree of anisotropy	1 = isotropic, >1 = anisotropic by definition; DA = length of longest divided by shortest mean intercept length vector	–
MIL	Mean intercept length	Measurements of structural anisotropy	–

**The variables highlighted in bold are the minimal set of variables that should be reported when describing trabecular bone morphology.**

The images can be used for interpretation much like a histological section, in comparing the structure to the known structure of bone. However, there are a number of advantages micro-CT has over other methods beyond conserving the specimen. It is significantly quicker and is less labor intensive than many other methods. A larger continuous region of interest can be examined than is possible using histological methods, where there is loss of sample with the preparation of each section. This results in a greater likelihood of capturing transition zones, such as the interface between material and old bone, to assess if there has been substantial integration of the new bone-scaffold construct and the existing bone. Furthermore, the 3D microarchitecture, as well as measurement such as volume and thickness, can be more faithfully represented by micro-CT as it does not rely on stereological methods to model these characteristics (Hildebrand and Beglinger, 1997).

Bouxsein et al. (2010) have suggested guidelines for the use of micro-CT when assessing bone microarchitecture. Further work has been done on using this data for the quantification of bone mineralization (Burghardt et al., 2008). The use of standardized protocols will aid the comparison of osteogenesis between different bioglass scaffolds and studies. This quantitative data is hugely useful for comparative studies and in reducing bias associated with subjective methods.

#### Angiogenesis

MicroCT scanning can make use of intravenous contrast media to visualize and measure the degree of angiogenesis. Usually, blood vessels have too low an x-ray attenuation to be visualized, which necessitates the use of contrast, of which there are two commonly used (MV-122—silicon rubber and BaSO_4_/gelatin). These allow for direct visualization of the vasculature and the calculation of various parameters from this data, though these are less well defined than in osteogenesis (Duvall et al., 2004). This method is akin to early use of agents to form casts of vascular structures such as those in the kidney, but with the benefit of using a more quantitative analytic tool to measure the outcome.

This technique is currently only used in post-mortem specimens as discussed below. It involves euthanasia of the specimen, followed by a multistep process of injecting the contrast medium and curing agent intracardially before a fixing period of several hours (Duvall et al., 2004). Following this the vasculature can be imaged, and various algorithms used to calculate the mentioned parameters.

*In vivo* imaging of angiography would mitigate the issues around destroying the specimen with the contrast agent and would represent a far superior imaging modality if the resolution and contrast could be upheld. Unlike contrast agents for *ex vivo* imaging which are static in the non-beating circulatory system, the contrast agents for *in vivo* use must have a different set of properties. These are uniquely different in murine models than humans due to the differences in animal heart rate and molecule size required relative to capillary foot processes. Continuous infusions may be required to adequately image the structure whilst the contrast is still *in situ*, due to the relatively fast first pass of the contrast if given as a bolus. Modified molecules for size and pegylation to avoid rapid extravasation and phagocytosis, respectively, are two other modifications required for *in vivo* vascular imaging such as in Fenestra VC (Badea et al., 2008).

#### Analysis

There are many reasons why microCT has become the commonest technique in evaluating bone morphology in this field. Being non-destructive, unlike histological approaches, allows serial scanning of the same specimen *in vitro*, without destroying the specimen and hence providing temporally sequential imaging, allowing the study of mineralization and structure change over a period of times in the same animal. This method of analysis can also be used in combination with the other techniques described (e.g., histological analysis at a final time point), allowing a greater data yield from a single specimen and reduces the number of animal specimens required.

In terms of the quality of data obtained, microCT provides comparable numerical results to traditional histomorphological techniques in both human and animal models. The benefit of microCT over these earlier methods in terms of accuracy is that microCT analyses a greater volume of interest (VOI), and the measurements are direct rather than inferred from single slices with assumptions made about structure by stereologic models. Furthermore, the vast number of output variables allows many parameters to be calculated from one scan. Although imaging requires some preparation of the specimen or anesthesia of the animal, it remains a quicker and more time-efficient technique than histological techniques or immunohistochemistry (Bouxsein et al., 2010).

When microCT imaging is compared to 2D histomorphic sections of the same specimen, measurements show high degrees of correlation, suggesting it is an accurate method for measuring various indices (Kuhn et al., 1990; Müller et al., 1998). MicroCT can also be used to image the scaffold of any manufactured biomaterial, whether implanted in bone in live animals or in prepared specimens. When compared to histological analysis of comparable specimens, contrast microCT produced similar estimations of blood vessel number but with the added benefit of 3D quantitative analysis (in an ischaemic hindlimb model of angiogenesis) (Duvall et al., 2004).

There are some downsides to microCT imaging in this context, such as the exposure of the animal to ionizing radiation. The exact significance of this is unclear but is more important with serial scanning of the same animal; with a potential to reduce bone growth by damaging stem cells as shown in one study (Klinck et al., 2008). Others argue that the effect is unlikely to be significant (Badea et al., 2008). Anesthesia of pre-clinical models is common, but lacks central protocols and carries risks such as death (Cicero et al., 2018). There is considerable heterogeneity in imaging protocols used by researchers, including scanner settings and the image analysis techniques. This has led to a call for more standardized protocols, and the publication of a standard set of data with each paper (Bouxsein et al., 2010). Unlike other approaches discussed in this paper, microCT does not provide information about cell types present, nor the biological activity of that tissue. However as mentioned previously it can be used in conjunction with methods that do give that information.

## Studying Gene Expression

The molecular mechanisms of osteogenesis and angiogenesis can be interrogated via measuring changes in the expression levels of related genes. The most popular method is quantitative reverse transcription polymerase chain reaction (RT-qPCR) where gene expression is measured against a reference gene. Real-time PCR measures PCR amplification as it occurs (using fluorogenic-labeled probes), allowing the real-time detection of specific amplification products and calculation of the starting concentration of nucleic acid. This contrasts with traditional PCR, where results are collected only after the reaction is complete, making it impossible to determine the starting concentration of nucleic acid. Two methods which are commonly used to quantify the results are the standard curve methods and the comparative threshold method. In the former, a standard reference curve is constructed from RNA of known concentration, which is then used to extrapolate quantitative information for mRNA targets of unknown concentrations. In the comparative threshold method, the Ct value of the sample is compared with a control and normalized to an appropriate endogenous housekeeping gene (where expression is assumed to be constant). This is also referred to as the 2^–ΔΔCt^ method (Livak and Schmittgen, 2001).

The genes of interest vary widely as the complex regulatory systems by which bone and blood vessel synthesis is controlled remains incompletely defined. In the case of bone formation, several intracellular signaling pathways are likely to be activated within progenitor cells, which modulate the levels of transcription factors involved in the osteoblast phenotype, which in turn regulate the genes that code for bone matrix proteins.

Osteogenesis-related genes can therefore include any implicated gene from regulators of the osteoblast phenotype, to various transcription factors and matrix proteins. Signaling cascades which promote osteogenic differentiation generally converge on two key transcription factors: proliferator-activated receptor-γ (PPARγ) and Runt-related transcription factor 2 (Runx2). PPARγ has well-described anti-osteoblastogenic effects while Runx2 is implicated in osteogenic regulation. Several cell signaling cascades are implicated (in multiple biological processes not exclusive to osteogenesis), such as β-catenin dependent Wnt signaling (as well as β-catenin independent signaling) (Taipaleenmaki et al., 2011; D’Alimonte et al., 2013), Hedgehog signaling (Fontaine et al., 2008; James et al., 2010), and NELL-1 (NEL-like protein 1) signaling (James et al., 2012). The families of angiogenic growth factors that have been described are equally extensive, including the vascular endothelial growth factors (VEGFs), fibroblast growth factors (FGFs), platelet-derived growth factors (PDGFs) and the angiopoietins (Ang-1 and Ang-2), and their roles in signaling cascades (Ferrara et al., 2003).

## Conclusion

The increasing burden of bone defects on an aging population necessitates further research into alternatives to conventional treatments. New bioactive glass compositions are constantly being developed and their properties can be altered by incorporating different elements into the glass composition. For example, Kargozar et al. (2019) reviews the effects of adding other ions in bioactive glasses such as strontium, cobalt (Kargozar et al., 2017; Kermani et al., 2020) or copper (Kargozar et al., 2021). Further assessment of bioactive glass constructs in inducing osteogenesis in osteoporotic models will aid in managing this demographic of patients.

As infection represents another significant cause of bone defects, further studies to investigate the effectiveness of these biomaterials should be tested under an infective burden. This research continue alongside the ongoing studies into the drug-releasing kinetics of bioactive glasses.

Bioactive glasses are also produced in forms ranging from powders to 3D scaffolds and research in ongoing in developing optimal constructs for tissue defects (Kargozar et al., 2018; Wang et al., 2019), The consideration of bioactive glass constructs in repairing a wider spectrum of tissues will, in particular, require assessing the biomaterial’s ability to induce angiogenesis. However, it will be necessary to consider additional methods to assess the effectiveness of the biomaterial in regenerating the target tissue.

With the expanding research and proposed applications for bioglasses, it is important to be able to consistently assess their capacity for osteogenesis and angiogenesis. This review article summarizes some of the common methods for assessing osteogenesis and angiogenesis and highlights the importance of these techniques in identifying plausible future bioactive glasses for clinical use.

## Author Contributions

WK and KS: conceptualization, funding acquisition, supervision, methodology. JC, AH, KS, and WK: investigation, writing. All authors contributed to the article and approved the submitted version.

## Conflict of Interest

The authors declare that the research was conducted in the absence of any commercial or financial relationships that could be construed as a potential conflict of interest.
